# Dual Carbon Goal-Based Quadrilateral Evolutionary Game: Study on the New Energy Vehicle Industry in China

**DOI:** 10.3390/ijerph20043217

**Published:** 2023-02-12

**Authors:** Tao Li, Lei Ma, Zheng Liu, Chaonan Yi, Kaitong Liang

**Affiliations:** 1 School of Intellectual Property, Nanjing University of Science and Technology, No. 200 Xiaolingwei Street, Xuanwu District, Nanjing 210094, China; 2 Centre for Innovation and Development, Nanjing University of Science and Technology, Nanjing 210094, China; 3 School of Business, Xianda College of Economics & Humanities Shanghai International Studies University, No. 390 Dong Tiyuhui Rd, Hongkou District, Shanghai 200083, China; 4 School of Public Affairs, Nanjing University of Science and Technology, Nanjing 210094, China; 5 Cardiff School of Management, Cardiff Metropolitan University, Western Ave, Cardiff CF5 2YB, UK

**Keywords:** Dual Carbon, new energy vehicle, innovation policy, quadrilateral evolutionary game

## Abstract

In an effort to tackle climate change, the “Dual Carbon” target raised by the Chinese government aims to reach peak carbon dioxide emissions by 2030 and to achieve carbon neutrality by 2060. Accordingly, policy incentives have accelerated the new energy vehicle (NEV) sector. Whilst previous studies have focused on the bilateral game between governments and manufacturers, NEV development has witnessed interaction among multiple players. In this paper, we construct a quadrilateral evolutionary game model, considering the impact of government policies, manufacturers’ R&D investments, dealers’ support, and consumer choice on the evolutionary stabilization strategy (ESS) in the context of China. The results show that: (1) in the absence of government incentives, there is no motivation for manufacturers, dealers and consumers to consider the development of NEVs; (2) government incentives affect manufacturers and consumers on the evolutionary paths in the short term. In the long term, benefit- and utility-based limited rationality has a dominant role in the ESS. This study contributes to the understanding of the multilateral dynamics of NEV innovation and provides important implications to practitioners and policy makers.

## 1. Introduction

Climate change has been a global challenge in recent years, with large amounts of CO_2_ emissions resulting in global warming, increasing the probability of climate extremes [1]. The impact is seen from various aspects including landscape ecology [2], biodiversity, and natural resources [3].

In order to tackle this challenge, a profound economic, technological and social institutional change is needed [4]. On a global scale, the urgency of considering new energy has been raised. According to the “World Energy Outlook 2022”, the demand for fossil energy is expected to peak within a few years based on existing national energy policies [5]. By September 2022, the carbon neutrality target covered 136 countries, affecting 83% of greenhouse gas emissions and 91% of GDP [6]. The energy crisis may become a turning point for clean energy development, as national energy policies continuously promote carbon reduction efforts. In 2021, the EU launched the “Fit for 55” plan, proposing twelve initiatives to reduce carbon emissions, which include energy, industry, transport, and buildings, and a commitment to a 55% reduction in greenhouse gas emissions by 2030 in comparison with 1990 [7]. The Chinese government announced the “Dual Carbon” target in 2020, aiming to “reach peak carbon emissions by 2030 and achieve carbon neutrality by 2060” [8]. This was followed by the “Action Plan for Carbon Dioxide Peaking Before 2030” and the “1+N” policy system, both issued by the Chinese government [9]. In the US, “The Long-term Strategy of the United States: Pathways to Net-Zero Greenhouse Gas Emissions by 2050” was released, pledging to reduce emissions by 50–52% by 2030 in comparison with 2005, and to achieve carbon neutrality by 2050 [10]. Other countries such as the UK, Russia, Japan and South Korea also proposed carbon neutral action plans. The carbon dioxide emission allowances were introduced by the EU, and accordingly, companies or individuals should eliminate their carbon footprint by purchasing carbon credits.

With policy support and technological advancements, green transformation is taking place worldwide. Among the efforts is the acceleration of the new energy vehicle (NEV) industry, which fundamentally restructures the traditional automobile industry. Three main players contribute to NEV innovation: traditional vehicle manufacturers, emerging NEV companies, and technology giants [11]. At present, the NEV power source mainly relies on the LiFePO_4_ battery energy storage. Limitations in charging efficiency and infrastructure lead to the weak market competitiveness of pure electric vehicles. In response to this, there are policy incentives to improve the competitiveness and popularity of pure electric vehicles (EVs) [12]. 

The NEV sector has witnessed significant growth in China. In 2022, China’s NEVs showed 7.058 million production units and 6.887 million unit sales: an increase of about 95% in comparison with 2011, meaning that China has maintained the global first position in the NEV market for eight consecutive years [13]. In fact, China’s global market share increased to 25.6% in 2022 with 679,000 units exported, accounting for about 10% of total sales [13].During the growth of the NEV industry, it is seen that manufacturers provide the technologies and the production, while dealers approach the market. Another important driver of market expansion comes from consumers [14]. Meanwhile, governments can utilize policy instruments to incentivize vehicle manufacturers to develop NEVs and to guide consumers to purchase the products. Whist existing studies provide valuable insights into the effects of policies for green technology innovation and market expansion of NEVs, they mainly focus on governments and manufacturers; whereas the roles of other players are yet underexplored. 

In this paper, we aim to propose a new model addressing the interconnection of governments, consumers, manufacturers, and dealers on an integral basis by exploring the NEV sector in China. The study makes theoretical and practical contributions from the following novel aspects. Compared with previous studies on carbon neutrality that used two-player and three-player evolutionary games, this study adopts a four-player evolutionary game, which is a new method. Additionally, this study analyzes the role of government incentives on manufacturers, dealers and consumers on a long-term time scale and at a macro level, which provides a different perspective. The paper also points out several evolutionary paths and explores the limitations of government incentives in the long run. 

The remainder of the paper is structured as follows: after this introduction, Section 2 reviews literature on the roles of manufacturers, markets, and governments in the NEV industry, alongside the application of game theory in this industry. Subsequently, the research gaps are identified. Section 3 elaborates the model and results. Section 4 further compares the findings with existing studies, which is followed by conclusions and a future research agenda.

## 2. Literature Review

The literature review covers four areas: green transformation from the manufacturer perspective, market concerns in the NEV industry, government policies and incentives, and game theory application.

### 2.1. Green Transformation from the Manufacturer Perspective

Green transformation is an emerging theme in the area of innovation due to increasing concern about the environment. Among the various topics, the issue of automotive carbon emissions has received long-term attention [15]. Studies utilizing modelling are seen in terms of carbon emission reduction methodology and green innovation from the manufacturer perspective. 

It is believed that carbon emission reduction methodology advocates for the introduction of operational algorithms to achieve carbon reductions in the short term, given policy incentives and green innovation [15]. For instance, artificial intelligence algorithms can help predict vehicle carbon emissions, as seen in a novel estimation method based on multifactor macro-fundamental diagrams for full sample traffic volume and overall road network emissions, to achieve road network carbon emission estimations [16]. Another model focuses on the achievement of carbon neutrality, targeting for the replacement of medium and large passenger cars with pure electric vehicles in Seoul [17]. Through the analysis of individuals’ choices of pure EVs, Li et al. find that driving cost, charging efficiency, and convenience of charging stations are the main influencing factors of NEV consumption [18]. Luo and Qiu propose a reservation mechanism to enhance the infrastructure utilization of public charging stations [19]. Wang et al. simulate a “Dual Carbon” pathway, which demonstrates a possible contribution of China’s practice to global emissions reduction [20].

Meanwhile, green innovation can provide the technological foundations to achieve the “Dual Carbon” goal. There are three dimensions of green innovation in SEM (structural equation modeling)-related research: green product innovation, recycling, and green publicity [21]. Li et al. measured the contribution of green innovation efficiency to “Dual Carbon” at a city level [22]. It was found that the induced effect of tariff schemes on green patents is verified, although their quality remains low [23]. Green innovation is constrained by the path dependence of energy, and electrical energy is still the main source of power for NEVs at present. The adoption of pure electric vehicles can reduce carbon emissions and PM2.5 with public health benefits, however, thermal power generation as the main source of energy supply still generates significant carbon emissions [24]. In the context of China, based on the carbon emission data for the post-industrial innovative manufacturing metropolis of Shenzhen, Liao et al. find that electricity consumption significantly contributes to the increase in carbon emissions [25]. Since the manufacturing and energy mix in Shenzhen has been upgraded to low carbon levels, the marginal impact of restructuring on CO_2_ emissions is not significant [25]. Unclean power sources are a major reason for reconsidering and restructuring plans to produce and use NEVs [26].

### 2.2. Market Concern in the New Energy Vehicle Industry

Alongside green innovation, environmental protection can limit the market acceptance of NEVs. From a consumer aspect, Boguslavsky, Sharov and Sharova find that the use of green energy may not be eco-friendly, mainly due to the environmental pollution caused by the storage of electrical energy usually with the help of lithium compounds [27]. Moreover, the increase in demand for lithium batteries in pure EVs triggers the battery end-of-life problem [28]. There is a lack of relevant international standards to make uniform regulations on the management of used lithium batteries, while the extension of the manufacturers’ responsibility increases the after-sales cost [29]. Zhu and Chen highlight that the demand for NEVs in China is related to power structures [30]. Although waste LiFePO_4_ batteries are treated by neutralization and landfill in China, the process of treating waste LiFePO_4_ batteries requires continuous research [30]. 

Safety is another consumer concern, as the use of lithium iron phosphate compounds is a limitation of pure EVs [31]. Furthermore, the carcinogenic and highly toxic compounds released by the ignition of lithium batteries in pure EVs can pose a threat to human health [31]. The risk of electromagnetic radiation from pure EVs has also raised research concerns. Through the observation of human driving reaction time tests to assess changes in neuropsychological cognitive function at a local static magnetic field (SMF) of 350 μT, He at el. find that participants’ driving cognitive function receives little effect with a given SMF intensity [32]. However, there is an increased risk of electromagnetic radiation from pure EVs in the real environment. A study for the long-term detection of Extremely Low Frequency Magnetic Field (ELFMF) in pure EVs reveals that regular maintenance does not reduce magnetic flux density, while major repairs or accidents may lead to stronger ELFMF exposure [29]. It is also noted that the low driving noise of pure EVs is not easily perceived by pedestrians and may create traffic safety problems [33,34].

Dealers are another important NEV stakeholder alongside consumers, governments, and vehicle manufacturers [35]. Through a survey to California Tesla retail stores, Cahill, Davies-Shawhyde & Turrentine find that dealers’ marketing practices and after-sales support have a significant impact on the market diffusion of innovative products such as NEVs [36]. In particular, dealers’ technical orientation, willingness to sell and demonstrate knowledge of EVs are the main factors contributing towards possible purchase intentions [37]. Another survey indicates that too much focus on policy incentives leads to a singular focus on promoting NEVs, while the inherent drawbacks of pure EVs and cheap used cars are barriers to NEV adoption [38].Moreover, dealers’ sales strategies and consumption trends are influenced by government and industry signals [39].

### 2.3. Government Policies and Incentives

In general, innovation policy tools can be divided into supply-side and demand-side instruments [40,41]. Supply-side policies aim to generate knowledge [41]. This is seen in terms of R&D, funding, tax incentives, risk investment, personnel training, improving research centers, infrastructure, and establishing industry clusters. On the other hand, policies aiming to promote consumption and accelerate innovation diffusion are regarded as demand-side instruments [40]. Accordingly, three policy frameworks have evolved. Frame 1 addresses both supply-side and demand-side. Frame 2 encourages networks and interactions among innovative actors. Frame 3, as an emerging framework, highlights sustainability and social changes [42]. 

It is believed that policies can stimulate the new energy R&Ds, guiding NEV consumption and industrial upgrading. Tu et al. measure the impact of a carbon emissions tax on the economic system using a four-sector dynamic stochastic general equilibrium model [43]. Chen et al. discuss the effect of a carbon emission trading system (ETS) using the Differences-in-Differences method (DID) and find that ETS has regional heterogeneity [44]. Meanwhile, Shen, Lin and Cheng explore the impact of a mixed incentive policy of carbon tax and carbon trading on a firm’s low carbon transition [45]. Carbon emissions trading is a market-based instrument. Using DID, Weng et al. find that carbon trading significantly reduces PM2.5 concentrations [46]. Based on the panel data of 11 provinces and cities in China’s Yangtze River Economic Belt from 2007 to 2019, green finance has played the part of an intermediary role in energy development [47]. 

Policies also influence demand and infrastructure. Fan and Shen model the effect of consumer preference on the proliferation of pure EVs [48]. The results from a survey of 1039 pure EV consumers indicate that just under one-third of consumers have a good understanding of government incentives, and more than half purchase pure EVs without such knowledge [49]. To promote cross-regional low-carbon development, there is a need to accelerate energy-saving and emission reduction technology R&D [50]. Additionally, it is essential to transform and upgrade industrial structures, alongside optimizing regional development and industrial transfer patterns [50]. To explore the impact of using pure EVs on logistics companies, Liao, Liu and Fu suggest that changes in carbon quotas are not related to delivery plans and corporate transformation, and that battery capacity and charging rates have a significant impact on the total cost of using electric vehicles [51].

Whilst policy incentives for NEV technologies have been studied in recent years, the focuses are either the supply side, namely the impact of tax, subsidy and licensing restriction policies on the proliferation of pure EVs [52], or the demand side, referring to the role of consumer preferences [48]. In addition, studies are concerned with the impact of subsidies, travel restrictions and infrastructure development on vehicle manufacturers’ R&D investment in pure EV technologies [53]. The impact of government subsidies on NEV development under different network structures has also been explored [54].

### 2.4. Game Theory Application

As NEV development requires interaction among multi-sided players, game theory has been applied to this industry, mainly in terms of NEV diffusion, supply side, and carbon reduction policies. 

Using an evolutionary game model to discuss NEV diffusion in complex networks of different sizes, Li, Jiao & Tang find that both sides of subsidy policies have a facilitating effect, while the supply-side subsidy has a better diffusion effect than the demand-side [52]. Similar results are obtained in a small-world network model [53]. A further study on social network games finds that: high subsidy rates and network size expansion lead to high diffusion; and that subsidy threshold rises with network size; and that large-scale and scale-free networks are more sensitive to subsidy rates [54]. However, the long-term effect of policies is not discussed [54]. Liao et al. establish a competitive game between internal combustion engine vehicle (ICEV) manufacturers and NEV manufacturers [55]. The results show that government subsidies have a boosting effect on the NEV market, but neither supply-side subsidies nor consumer-side subsidies can improve the profitability of firms [55]. Han et al. explore the mechanism of policy transition on the evolution of R&D strategies in collaborative innovation networks for NEV [56]. Zhu et al. analyze the impact of license plate restriction (LPR) policies on NEV diffusion using a two-stage Stackelberg game [57].

In terms of the supply-side, Ma et al. use a Stackelberg game to reveal the negative impact of policies on the NEV supply chain system of vendors and customers, where policies lead to uncontrollability of the supply chain system [58]. Wang, Xu and Zhu study the carbon reduction decision and profit maximization between manufacturers and supply chain members using a differential game [59]. Haghighi et al. simulate the game between Generation Expansion Planning (GEP) and government subsidies for carbon emission reduction from power stations, and perform a sensitivity analysis to provide a theoretical basis for the energy supply problem of NEVs [60]. The problem of constructing a Stackelberg game for real-time equilibrium power markets is investigated [61]. Additionally, the design of reward and punishment mechanisms for power battery recycling is explored from a supply-side perspective. The equilibrium decisions of firms are identified in a closed-loop NEV battery supply chain, and the combined effects of government subsidy policies and cost-sharing coordination mechanisms is discovered from a dynamic perspective [62]. This is an important guide for coordination and cooperation among firms in a closed-loop supply chain, the decisions of firms, and the formulation of government subsidy policies [62].

Game theoy is also applied in carbon reduction policies-related research. Wei theoretically analyzes carbon tax policy choices with a sequential game [61]. Geng, Ji and Yang use an evolutionary game to discuss the mechanism by which a coalition of firms and governments are jointly responsible for corporate carbon reduction efforts [62]. Hussain and Lee develop a duopoly game model to analyze the role of green bond provision, emission taxes and emission allowance [63]. Kim et al. analyze the impact of loan support and carbon taxes, concluding that additional R&D budgets should be used to enhance green technologies [64]. Based on a trilateral evolutionary game model with high and low efficiency groups and local governments, respective strategic choices of the players are discussed [65]. It is found that government subsidies are critical to alleviate the funding shortage of the NEV industry [66]. Zhan and Zhou et al. combine game theory and reinforcement learning to investigate the evaluation and strategy optimization methods of government subsidy strategies under various situations [66]. The strategic choices of government subsidies, firms’ production, and consumers’ purchasing behaviour are further analyzed in the context of subsidy withdrawal [67]. Huang et al. explore incentive policies for hydrogen fuel cells and highight protectionism and divergent interests among different levels of governments and companies [67]. Through an evolutionary game considering governments, charging infrastructure operators, real estate agencies, and NEV users, the business model of NEV charging infrastructure investment and operations are evaluated [68]. Zhao, Ma and Wang use an evolutionary game to analyze the effect of government policies on motivating cold chain logistics firms to adopt NEVs [69]. Fan et al. develop an evolutionary game model, finding that green innovation incentives, environmental tax and innovation subsidies are effective policy tools to promote green innovation diffusion [70]. However, interfirm competition and alliances can make incentives ineffective [71]. In fact, the strength of incentives can inhibit green innovation, where firms can adopt a conservative attitude in the face of green innovation [71].

It is noted that sequential game, Stackelberg game, differential game, and evolutionary game are used in NEV-related studies. They present not only the NEV policy effects but also the evolution of the game equilibrium of the players. Nevertheless, existing studies consider mainly two or three players. They do not reflect the complex interaction in the NEV market. In fact, government policies alone cannot fully promote NEV development, whether tax and subsidy policies, or licensing plate restrictions and NEV purchase subsidies. 

### 2.5. Identification of the Research Gaps

Based on the above literature review, there are research gaps. Few studies have considered governments, manufacturers, dealers and consumers simultaneously as stakeholders when constructing the evolutionary game model. Although the literature has discussed various types of incentives, such as carbon tax, carbon trading, and carbon credits, the purpose of policies is to stimulate manufacturers to invest in R&D and to stimulate consumer acceptance of new energy technologies. Especially in the circumstance of complex economic environment and long-term effects, policies are often crossed or implemented in parallel for policy makers, and policy evaluation is ultimately conducted in terms of costs and benefits from a macro perspective. 

In fact, the equilibrium of NEV incentives under governments, manufacturers, dealers and consumer participation and their practical implications are yet to be explored. Thus, this study attempts to fill these research gaps. Specifically, we place the four participants of governments, manufacturers, distributors and consumers in a unified framework; we construct a quadrilateral evolutionary game; we consider government incentives, manufacturers’ R&D, dealers’ marketing support and consumers’ decisions; and we discuss the ESS. 

## 3. Model and Results 

Evolutionary game model is widely used in ecological governance, decision analysis and other fields. The advantage is to reveal the process of different groups reaching a stable state in long-term competition from a dynamic perspective, and to place more emphasis on the finite rationality of game participants. The formation and evolution of the NEV market is a manifestation of different combinations of game strategies between groups, in line with the basic idea of evolutionary game model [55]. Researchers have already identified dealers’ marketing practices, technical orientation, willingness to sell, and demonstrated knowledge of EV as main factors contributing to possible purchase intention [35]. 

However, recent studies focusing on a two-player or three-player game among governments, consumers and manufacturers [57,65,67,68,70] have limitations. 

To make the research more practical, we construct a quadrilateral evolutionary game model. Among them, governments, manufacturers, dealers and consumers are the four groups of limited rationality pursuing their own interest maximization in this model. This approach is justified by considering the introduction of carbon neutrality by governments [5,7,8,10] and the prevalence of dealership patterns in the automotive industry in both developed and developing countries [36]. In the NEV market structure, governments are the regulator of carbon emission reduction, manufacturers are the developers and producers of NEVs and ICEVs, dealers play the role of product and policy diffusion, and consumers test policy effects by actions. These four players raise complexity and uncertainty. Hence, hypotheses are developed as follows.

**Hypothesis 1.**  *Each player has limited rational, which means each player cannot fully grasp all information of the other players. Players can gradually determine the optimal strategy by repeatedly trying and observing.*

**Hypothesis 2.**  *According to the literature* [35]*, participants in the NEV market include governments, manufacturers, dealers, and consumers.*

**Hypothesis 3.**  *According to the literature* [55,58,68,70]*, the effect of the dual carbon policies on participants in the NEV market is non-linear in the long run. In other words, policy X produces effect Y in the short run, but in the long run, effect Y is time-varying.*

### 3.1. Premise and Notation

**Strategies for governments**. Governments’ strategy set is Incent and Not Incent, denoted as **{I, NI}**. The probability of government selecting **{I}** is denoted as x. Incentives include tax reduction and subsidies for the end market, prompting manufacturers and consumers to consider NEVs with low carbon emissions. The government administrative cost incurred by the incentive is denoted as $C_{1}$. Incentives lead to gains in environmental protection denoted as $R_{1}$, and improvement in government performance is denoted as $I_{g}$. Additional government tax is denoted as $R_{2}$ on manufacturers who do not invest R&D in NEVs. Dealers who do not promote new energy are subject to additional tax from governments, which is denoted as $R_{3}$. The administrative cost is $\beta \cdot C_{1},\beta \in \left(0,1\right)$ when governments choose **{NI}**. Meanwhile, the benefits of environmental protection are reduced to $\alpha R_{1},\alpha \in \left(0,1\right)$, and the loss of government performance is denoted as $D_{g}$.

**Strategies for manufacturers**. The manufacturers’ strategy set is R&D in NEVs or Hold, denoted as **{RD, H}**. The probability of selecting **{RD}** is denoted as y. The brand licensing revenue paid by dealers is denoted as $R_{4}$. If manufacturers choose **{RD}** and dealers provide weak support (denoted as **{WS}**) then dealers receive penalty $C_{3}$. Manufacturers’ cost of R&D is $C_{2}$. Consumers’ brand stickiness is increased to $I_{e}$ while consumers select **{NEV}** and manufacturers choose **{RD}**. The cost incurred by manufacturers for selecting **{Hold}** (denoted as **{H}**) is $\gamma \cdot c_{2},\gamma \in \left(0,1\right)$. If manufacturers choose **{Hold}** and consumers select **{NEV}**, the brand stickiness will be decreased to $D_{e}$.

**Strategies for dealers**. Dealers provide support for products with a strategy set of strong support and weak support, denoted as **{SS, WS}**. The probability of selecting strategy **{SS}** is z. If dealers choose **{SS}**, this will result in a market gain of $R_{5}$ and manufacturers’ incremental rating of $I_{d}$. If dealers are short-term profit-seeking and choose **{WS}** by deciding the level of service, the market gain is obtained as $\delta \cdot R_{5},\delta \in \left(1,\infty \right)$; the loss of rating is $D_{d}$; and the cost of providing claims to consumers is L.

**Strategies for consumers**. Consumers’ strategy set is **{NEV, ICEV}**. The probability that consumers choose **{NEV}** is w. If dealers choose **{SS}**, consumers’ product satisfaction will be increased by $I_{c}$>. Otherwise the satisfaction will be decreased by $D_{c}$. The lower technological maturity of NEV leads to more quality claims, and the cost associated with consumers choosing **{NEV}** and incurring a claim is $C_{4}$, while the value of the claim is L. If manufacturers choose **{Hold}** and dealers select **{Strong Support}**, consumers choosing **{NEV}** will seek additional compensation Q.

It is assumed that when governments choose **{I}** or manufacturers choose **{RD}**, dealers’ choice of **{WS}** is less profitable than **{SS}**. The condition is satisfied via the following inequality set.

(1) $$\left\{\begin{array}{c} I_{d}+C_{3}+D_{d}+\left(1-\delta \right)R_{5}>0\\ I_{d}+R_{3}+D_{d}+\left(1-\delta \right)R_{5}>0 \end{array}\right.$$

Government incentives to manufacturers who invest in NEV R&D lead to a reduction in government benefits. This condition is satisfied as follows.

(2) $$R_{1}-C_{1}<\alpha R_{1}-\beta C_{1}$$

The symbols and descriptions are shown in Table 1.

### 3.2. Dynamic Game and Payoffs

**Figure 1:**
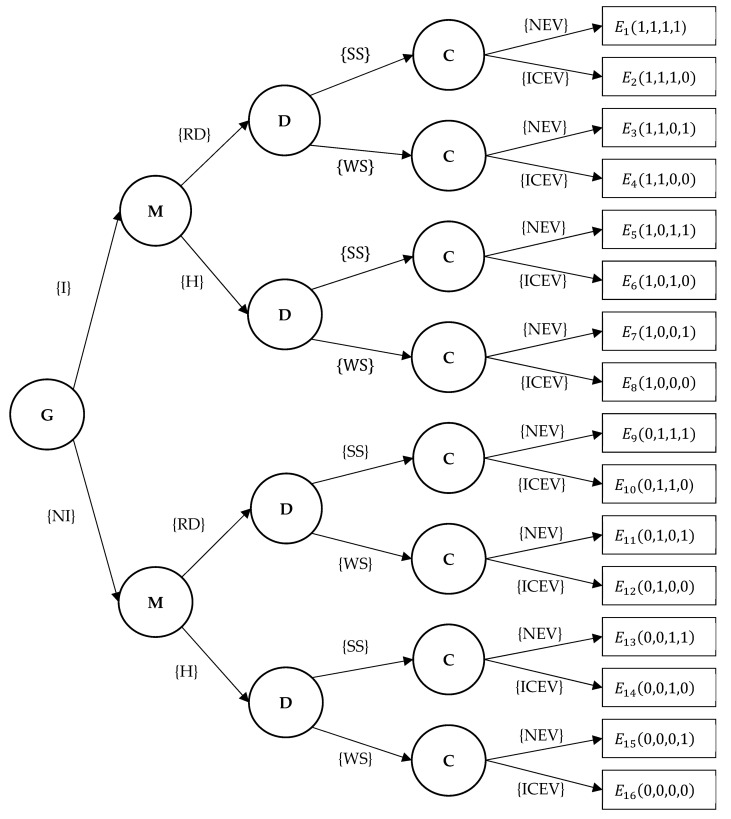
The dynamic game and its strategy combination.

The strategy choices of governments, manufacturers, dealers and consumers constitute a dynamic game that generates 16 strategy combinations (Figure 1) and a payoff matrix (Table 2).

### 3.3. Replicator Dynamics Equations

#### 3.3.1. RDE for Governments

The expected return on governments’ choice of **{I}** has the following form.

(3) $$E_{gx}=-C_{1}+R_{1}+\left(1-y\right)\left(R_{2}+R_{3}+wI_{g}-zR_{3}\right)$$

The expected return on governments’ choice of **{NI}** is as below.

(4) $$E_{gnx}=-w\left(1-y\right)D_{g}+\alpha R_{1}-\beta C_{1}$$

The expected return on governments’ choice between **{I}** and **{NI}** is as below.

(5) $$E_{g}=xE_{gx}+\left(1-x\right)E_{gnx}$$

The dynamic replication equation for governments is as below.

(6) $$\varphi \left(x\right)=\frac{dx}{dt}=x\left(E_{gx}-E_{g}\right)=\left(1-x\right)x\left(\left(1-\alpha \right)R_{1}-\left(1-\beta \right)C_{1}+\left(1-y\right)\left(R_{2}+\left(1-z\right)R_{3}+w\left(D_{g}+I_{g}\right)\right)\right)$$

#### 3.3.2. RDE for Manufacturers

The expected return on manufacturers’ choice of **{RD}** is as follows.

(7) $$E_{my}=-C_{2}+C_{3}+R_{4}+wI_{e}-zC_{3}$$

The expected return on manufacturer’s choice of **{H}** is as follows.

(8) $$E_{mny}=R_{4}-wD_{e}-xR_{2}-\gamma C_{2}$$

The expected return on manufacturer’s choice between **{RD}** and **{H}** is as follows.

(9) $$E_{m}=yE_{my}+\left(1-y\right)E_{mny}$$

The dynamic replication equation for manufacturers is shown below.

(10) $$\phi \left(y\right)=\frac{dy}{dt}=y\left(E_{my}-E_{m}\right)\;=\left(1-y\right)y\left(\left(1-z\right)C_{3}+w\left(D_{e}+I_{e}\right)+xR_{2}-\left(1-\gamma \right)C_{2}\right)$$

#### 3.3.3. RDE for Dealers

The expected return on dealers’ choice of **{SS}** is as follows.

(11) $$E_{dz}=-R_{4}+R_{5}+wD_{d}\left(1-x\right)\left(1-y\right)+I_{d}\left(x+y-xy\right)$$

The expected return on dealers’ choice of **{WS}** is as below.

(12) $$E_{dnz}=-R_{4}+\left(-R_{3}+R_{3}y-wL\right)x-C_{3}y+wL\left(-1+x\right)y-D_{d}\left(w+x-wx+\left(-1+w\right)\left(-1+x\right)y\right)+R_{5}\delta$$

The expected return on dealers’ choice between **{SS}** and **{WS}** is as below.

(13) $$Ed=zE_{dz}+\left(1-z\right)E_{dnz}$$

The dynamic replication equation for dealers is elaborated as below.

(14) $$\psi \left(z\right)=\frac{dz}{dt}=z\left(E_{dy}-E_{d}\right)\;=\left(-1+z\right)z\left(-\left(\left(I_{d}+R_{3}+wL\right)x\right)-\left(C_{3}+I_{d}+wL-\left(I_{d}+R_{3}+wL\right)x\right)y-D_{d}\left(x+2w\left(-1+x\right)\left(-1+y\right)+y-xy\right)+R_{5}\left(-1+\delta \right)\right)$$

#### 3.3.4. RDE for Consumers

The expected return on consumers’ choice of **{NEV}** is as below.

(15) $$E_{cw}=-C_{4}+D_{c}\left(-1+z\right)+\left(I_{c}+Q\right)z+\left(-x+\left(-1+x\right)y\right)\left(-L+\left(L+Q\right)z\right)$$

The expected return on consumers’ choice of **{ICEV}** is as below.

(16) $$E_{cnw}=\left(-1+z\right)D_{c}+zI_{c}$$

The expected return on consumers’ choice between **{NEV}** and **{ICEV}** is as below.

(17) $$E_{c}=zE_{cw}+\left(1-z\right)E_{cnw}$$

The dynamic replication equation for consumers is developed as below.

(18) $$\omega \left(w\right)=\frac{dw}{dt}=w\left(E_{cw}-E_{c}\right)\;=\left(-1+w\right)w\left(C_{4}-zQ+\left(x-xy+y\right)\left(\left(-1+z\right)L+zQ\right)\right)$$

### 3.4. Evolutionary Stabilization Strategy (ESS) and Stability Analysis

The replicated dynamic system and its stabilization strategies are obtained through the joint Equations (6), (10), (14) and (18).

(19) $$\left\{\begin{array}{c} \varphi \left(x\right)=\frac{dx}{dt}=x\left(E_{gx}-E_{g}\right)\\ \phi \left(y\right)=\frac{dy}{dt}=y\left(E_{my}-E_{m}\right)\\ \psi \left(z\right)=\frac{dz}{dt}=z\left(E_{dy}-E_{d}\right)\\ \omega \left(w\right)=\frac{dw}{dt}=w\left(E_{cw}-E_{c}\right) \end{array}\Rightarrow \begin{array}{c} x=0,x=1\\ y=0,y=1\\ z=0,z=1\\ w=0,w=1 \end{array}\right.$$

Equation (19) shows that pure strategies of governments, manufacturers, distributors and consumers are their own evolutionary stable strategies.

According to the Lyapunov dynamic stabilization method [72] to determine the stable strategy combinations of Equation (19), the stable solution of a multigroup evolutionary game is a pure strategy Nash equilibrium [73] when: (a) the eigenvalues of the Jacobian matrix corresponding to the strategy combination are less than zero; or (b) the Jacobian matrix of the strategy combination satisfies both $det\left(J\right)>0,tr\left(J\right)<0$. We construct Jacobian matrix (Equation (20)) for Equation (19) and perform stability analysis as shown below.

(20) $$J=\left|\begin{array}{cccc} \frac{\partial \varphi \left(x\right)}{\partial x} & \frac{\partial \varphi \left(x\right)}{\partial y} & \frac{\partial \varphi \left(x\right)}{\partial z} & \frac{\partial \varphi \left(x\right)}{\partial w}\\ \frac{\partial \phi \left(y\right)}{\partial x} & \frac{\partial \phi \left(y\right)}{\partial y} & \frac{\partial \phi \left(y\right)}{\partial z} & \frac{\partial \phi \left(y\right)}{\partial w}\\ \frac{\partial \psi \left(z\right)}{\partial x} & \frac{\partial \psi \left(z\right)}{\partial y} & \frac{\partial \psi \left(z\right)}{\partial z} & \frac{\partial \psi \left(z\right)}{\partial w}\\ \frac{\partial \omega \left(w\right)}{\partial x} & \frac{\partial \omega \left(w\right)}{\partial y} & \frac{\partial \omega \left(w\right)}{\partial z} & \frac{\partial \omega \left(w\right)}{\partial w} \end{array}\right|.$$

The analysis of Table 3 shows that there are two ESSs: $E_{6}\left(1,0,1,0\right)$ and $E_{16}\left(0,0,0,0\right)$.

The strategy combination for the first ESS point $E_{6}\left(1,0,1,0\right)$: government chooses **{I}**, manufacturer chooses **{H}**, dealer chooses **{SS}**, consumer chooses **{ICEV}**. The environmental benefits and government performance from government strategy **{I}** are greater than the benefits from strategy **{NI}**. Manufacturer and dealer are limited rational and their strategies are driven by profits. Regardless of how much sunk cost manufacturer invested in R&D of NEVs, after government selecting **{I}**, manufacturer will wait for the market response. Thus **{H}** is a stable strategy for manufacturer. Dealer prefers marketing and aftersales support for products favored by consumer, and thus **{SS}** is a stable strategy for dealer. Limited rational consumer considers technical maturity and convenience of NEVs. Therefore **{ICEV}** is the stable strategy. Hence the equilibrium of this noncooperative dynamic game evolves to the stabilization point $E_{6}\left(1,0,1,0\right)$, i.e., the strategy combination **{I × H × SS × ICEV}**, where the social welfare of the four game participants is maximized.

The strategy combination for the second ESS point $E_{16}\left(0,0,0,0\right)$: government chooses **{NI}**, manufacturer chooses **{H}**, dealer chooses **{WS}**, and consumer chooses **{ICEV}**. Under this strategy combination, the cost of government strategy **{I}** outweighs the benefit, which will result in **{NI}** as the stable strategy. With no government incentives for NEV, limited rational consumer measures the utility of the vehicle in terms of price, charging facility input cost, convenience, value retention and replacement cost, hence chooses **{ICEV}** as the stable strategy. With government selecting **{NI}** and consumer selecting **{ICEV}**, manufacturer has no profit drivers to invest in NEV R&D. The high level of technological maturity allows fuel vehicles to generate stable cash flow, while maximizing profits by cutting marketing and supporting costs for dealers.

## 4. Conclusions

Related studies have provided valuable insights into the effects of policy incentives on green technology innovation and market expansion of NEVs. However, consumers, manufacturers, dealers and governments should be considered on an integral basis. Whilst most studies have selected only two types of actors to discuss the role of a factor on the NEV market expansion, our study has filled the research gaps. Specifically, we have considered China’s NEV sector, placing the four actors of governments, manufacturers, dealers and consumers in a unified framework of carbon emission reduction environmental governance. We have constructed a quadrilateral evolutionary game, considering government incentives, manufacturer R&D, dealer marketing support and consumer decisions, and discussed the ESS. The findings and implications can be summarized from the following aspects.

Firstly, policy incentives play an important role in the promotion of NEVs. Since pure electricity is still the main source of power for NEVs, technological limitations drive limited rational consumers to purchase ICEVs. Policy incentives can create a certain level of market demand for NEVs, and dealers will engage this market demand to provide strong marketing and product support. This result supports the findings from Zarazua De Rubens, Noel, et al. [36,37]. Additionally, the goal of dealers is not only to achieve sales targets and earn profits. Instead, governments should incentivize manufacturers and dealers to collaboratively collect market information and complete NEV proliferation programs. NEV’s green technology iteration and upgrade is an inevitable trend. The automotive market is becoming more and more competitive, and the channel resources available to each manufacturer are decreasing. Incentives for manufacturers and dealers to promote intra-industry synergy are conducive to consolidating the relationship among manufacturers, dealers, consumers and governments. This is an important guarantee for gaining policy advantage in regional markets.

Meanwhile, government policy incentives can be considered to reduce or waive taxes on NEVs, reduce or waive vehicle right-of-way fees, subsidize the installation of charging facilities under individual ownership, improve the retention of public charging facilities, and use price mechanisms to improve the flow rate of public charging facilities. In general, NEVs with bi-directional subsidies are still more expensive than ICEVs, while NEV energy supply methods lack technological innovation. NEV manufacturers such as Tesla, BYD, and NIO continue to improve their public charging facilities and battery life to attract consumers. In the meantime, traditional automakers in Europe, America, and Japan are focusing on cleaner hydrogen R&D. There is protectionism and divergent interests among different levels of governments and companies [67]. Therefore, the incentive policies should balance the market expansion and green innovation of NEVs. In other works, there is a need to strike a balance between promoting the economic development of the NEV industry in the short term and guiding the green technology upgrade of NEVs in the long term. Policies can help break the geographical restrictions and geopolitics, integrating the global industry chain synergy model with NEV green innovation.

Furthermore, the process of bringing clean energy from research to market for civilian use is a long-term one. Subsidies and tax exemption incentives will only attract consumer interest in the short term. Based on our study, two ESSs $E_{6}\left(1,0,1,0\right)$ and $E_{16}\left(0,0,0,0\right)$ construct a long-term evolutionary path. When governments choose strategy **{I}** (Incent), the game evolves along with the path: $E_{8}\left(1,0,0,0\right)\to E_{4}\left(1,1,0,0\right)\to E_{2}\left(1,1,1,0\right)\to E_{1}\left(1,1,1,1\right)\to E_{2}\left(1,1,1,0\right)\to E_{6}\left(1,0,1,0\right)$. The other three participants’ positive reactions to government incentives forms the short term sub-evolutionary path $E_{8}\to E_{4}\to E_{2}\to E_{1}$. This confirms the findings in the literature [45,48,55,73]. The remaining sub-evolutionary path $E_{1}\to E_{2}\to E_{6}$ corroborates that incentives are unsustainable in the long run. This finding has extended previous studies with evolutionary game [55,58,68,70]. $E_{16}\left(0,0,0,0\right)$ is the other ESS where governments choose {NI} (No Incent); afterwards there is no motivation for manufactures, dealers and consumers to consider NEVs.

Another practical implication is that leading companies tend to have more patent achievements, capital and human resource advantages to carry out clean energy R&D. Furthermore, governments should be more concerned about whether monopolies are created in the patenting and marketization of new technologies, and introduce competition from diverse clean energy technology companies.

There are some limitations in this study. Future research can construct cost–benefit models for NEVs with different technologies, portraying the patent layout and technology path of NEV, and introducing a product life cycle framework to analyze the decision making of vehicles based on various new energy technologies.

## Figures and Tables

**Table 1:** List of Symbols.

Symbol	Descriptions
{I, NI}	Government strategy set {Incent, No Incent}
{RD, H}	Manufacturer strategy set {R&D, Hold}
{SS, WS}	Dealer strategy set {Strong Support, Weak Support}
{NEV, ICEV}	Consumer strategy set {New Energy Vehicle, Internal Combustion Engine Vehicle}
x	The probability of governments selecting strategy {I}.
y	The probability of a manufacturer selecting strategy {RD}.
z	The probability of a dealer selecting strategy {SS}.
w	The probability of consumers selecting {NEV}.
$C_{1}$	The cost of government administration brought about by strategy {I}
$R_{1}$	Benefits of environmental protection from strategy {I}.
$I_{g}$	Improved government performance brought by strategy {I}.
$R_{2}$	Additional taxes on manufacturers selecting strategy {H}.
$R_{3}$	Additional taxation when governments incent NEV but dealers choose {WS}.
β	The cost factor when governments choose strategy {NI}.
α	Benefit factor for environmental protection when governments choose {NI}.
$D_{g}$	Loss of performance when governments select strategy {NI}.
$R_{4}$	Brand licensing fees charged by manufacturers to dealers.
$C_{2}$	Cost incurred by manufacturers selecting strategy {RD}.
$C_{3}$	Penalty paid by dealers when manufacturers choose to {RD} and dealers select {WS}.
$I_{e}$	Increment of brand stickiness when manufacturers choose {RD} and consumers choose {NEV}.
γ	The cost factor when manufacturers choose strategy {H}.
$D_{e}$	The deduction of brand stickiness when manufacturers select strategy {H} and consumers choose {NEV}.
$R_{5}$	Profits from dealers selecting strategy {SS}.
$I_{d}$	Incremental ratings by dealers selecting strategy {SS}
δ	Revenue factor for dealers selecting strategy {WS}.
$D_{d}$	If dealers are short-term profit-seeking and choose {WS} by deciding the level of service, the market gain is obtained as $\delta \cdot R_{5},\delta \in \left(1,\infty \right)$, and the loss of rating is $D_{d}$
L	Value of consumers’ claim.
$I_{c}$	Increment of consumer satisfaction from dealer selecting strategy {SS}.
$D_{c}$	Deduction of consumer satisfaction from dealer selecting strategy {WS}.
$C_{4}$	Consumer cost of claims incurred by choosing strategy {NEV}.
Q	The additional compensation from consumers selecting {NEV} in terms of manufacturers choosing {H} and dealers choosing {SS}

**Table 2:** Payoff Matrix.

				Dealers			
				{SS}		{WS}	
				Consumer		Consumer	
				{NEV}	{ICEV}	{NEV}	{ICEV}
Governments	**{I}**	Manufacturer	**{RD}**	$R_{1}-C_{1}$, $I_{e}+R_{4}-C_{2}$, $R_{5}-R_{4}+I_{d}$, $I_{c}-C_{4}$	$R_{1}-C_{1}$, $R_{4}-C_{2}$, $R_{5}-R_{4}+I_{d}$, $I_{c}$	$R_{1}-C_{1}$, $I_{e}+R_{4}-C_{2}+C_{3}$, $\delta R_{5}-R_{4}-D_{d}-L-C_{3}$, $L-D_{c}-C_{4}$	$R_{1}-C_{1}$, $R_{4}-C_{2}+C_{3}$, $\delta R_{5}-R_{4}-D_{d}-C_{3}$, $-D_{c}$
			**{H}**	$R_{1}-C_{1}+I_{g}+R_{2}$, $R_{4}-R_{2}-D_{e}-\gamma C_{2}$, $R_{5}-R_{4}+I_{d}$, $I_{c}-C_{4}$	$R_{1}-C_{1}+R_{2}$, $R_{4}-R_{2}-\gamma C_{2}$, $R_{5}-R_{4}+I_{d}$, $I_{c}$	$R_{1}-C_{1}+I_{g}+R_{2}+R_{3}$, $R_{4}-R_{2}-D_{e}-\gamma C_{2}$, $\delta R_{5}-R_{4}-D_{d}-L-R_{3}$, $L-D_{c}-C_{4}$	$R_{1}-C_{1}+R_{2}+R_{3}$, $R_{4}-R_{2}-\gamma C_{2}$, $\delta R_{5}-R_{4}-D_{d}-R_{3}$, $-D_{c}$
	**{NI}**	Manufacturer	**{RD}**	$\alpha R_{1}-\beta C_{1}$, $I_{e}+R_{4}-C_{2}$, $R_{5}-R_{4}+I_{d}$, $I_{c}-C_{4}$	$\alpha R_{1}-\beta C_{1}$, $R_{4}-C_{2}$, $R_{5}-R_{4}+I_{d}$, $I_{c}$	$\alpha R_{1}-\beta C_{1}$, $I_{e}+R_{4}-C_{2}+C_{3}$, $\delta R_{5}-R_{4}-D_{d}-L-C_{3}$, $L-D_{c}-C_{4}$	$\alpha R_{1}-\beta C_{1}$, $R_{4}-C_{2}+C_{3}$, $\delta R_{5}-R_{4}-D_{d}-C_{3}$, $-D_{c}$
			**{H}**	$\alpha R_{1}-D_{g}-\beta C_{1}$, $R_{4}-D_{e}-\gamma C_{2}$, $R_{5}-R_{4}-D_{d}$, $I_{c}-C_{4}+Q$	$\alpha R_{1}-\beta C_{1}$, $R_{4}-\gamma C_{2}$, $R_{5}-R_{4}$, $I_{c}$	$\alpha R_{1}-D_{g}-\beta C_{1}$, $R_{4}-D_{e}-\gamma C_{2}$, $\delta R_{5}-R_{4}-D_{d}$, $-D_{c}-C_{4}$	$\alpha R_{1}-\beta C_{1}$, $R_{4}-\gamma C_{2}$, $\delta R_{5}-R_{4}$, $-D_{c}$

**Table 3:** Analysis of Evolutionary Stability of Equilibrium Points.

Equilibrium Point ^①^	$Det\left(J\left(E_{i}\right)\right)$ ^②^	$Tr\left(J\left(E_{i}\right)\right)$ ^③^	Result
$E_{1}\left(1,1,1,1\right)$	>0	>0	Instability Point ^④^
$E_{2}\left(1,1,1,0\right)$	>0	>0	Instability Point
$E_{3}\left(1,1,0,1\right)$	>0	>0	Instability Point
$E_{4}\left(1,1,0,0\right)$	>0	>0	Instability Point
$E_{5}\left(1,0,1,1\right)$	<0	Indeterminant ^⑦^	Saddle Point ^⑤^
$E_{6}\left(1,0,1,0\right)$	>0	<0	ESS ^⑥^
$E_{7}\left(1,0,0,1\right)$	>0	>0	Instability Point
$E_{8}\left(1,0,0,0\right)$	>0	>0	Instability Point
$E_{9}\left(0,1,1,1\right)$	<0	Indeterminant	Saddle Point
$E_{10}\left(0,1,1,0\right)$	<0	<0	Instability Point
$E_{11}\left(0,1,0,1\right)$	<0	Indeterminant	Saddle Point
$E_{12}\left(0,1,0,0\right)$	<0	Indeterminant	Saddle Point
$E_{13}\left(0,0,1,1\right)$	>0	>0	Instability Point
$E_{14}\left(0,0,1,0\right)$	<0	Indeterminant	Instability Point
$E_{15}\left(0,0,0,1\right)$	>0	>0	Instability Point
$E_{16}\left(0,0,0,0\right)$	>0	<0	ESS
Explanation of symbols: ① The dominant strategies combination for all players in the evolutionary game. ② The determinant of the Jacobian matrix of the equilibrium point. ③ The trace of the Jacobian matrix of the equilibrium point. ④ The state of the evolutionary game is not robust when small disturbances appear. ⑤ Saddle point is a singularity that is stable along a certain strategy direction and unstable along another strategy direction. ⑥ ESS is a strategy or group of strategies adopted by a population in a particular environment, which is non-penetrable, i.e., the population cannot be invaded by other strategies or groups of strategies that initially account for a small percentage ⑦ Unable to determine positive or negative direction.			

## Data Availability

The data that support the findings of this study are available on request from the corresponding author.
